# A Review on Radar-Based Human Detection Techniques

**DOI:** 10.3390/s24175709

**Published:** 2024-09-02

**Authors:** Muhammet Talha Buyukakkaslar, Mehmet Ali Erturk, Muhammet Ali Aydin

**Affiliations:** 1 Department of Computer Engineering, Faculty of Engineering, Istanbul University-Cerrahpasa, Istanbul 34320, Türkiye; aydinali@iuc.edu.tr; 2 Department of Computer Engineering, Istanbul University, Istanbul 34134, Türkiye; mehmetali.erturk@istanbul.edu.tr

**Keywords:** frequency-modulated continuous wave radars, human recognition, micro-Doppler radars, spoofing attacks

## Abstract

Radar systems are diverse and used in industries such as air traffic control, weather monitoring, and military and maritime applications. Within the scope of this study, we focus on using radar for human detection and recognition. This study evaluated the general state of micro-Doppler radar-based human recognition technology, the related literature, and state-of-the-art methods. This study aims to provide guidelines for new research in this area. This comprehensive study provides researchers with a thorough review of the existing literature. It gives a taxonomy of the literature and classifies the existing literature by the radar types used, the focus of the research, targeted use cases, and the security concerns raised by the authors. This paper serves as a repository for numerous studies that have been listed, critically evaluated, and systematically classified.

## 1. Introduction

Radar systems are diverse and used in industries such as air traffic control, weather monitoring, and military and maritime applications. Within the scope of this study, we focus on using radar for human detection and recognition. These radars are typically used in automobiles, healthcare, and security applications. While they can be used in various fields, such as human motion recognition, security, and surveillance systems to care for the elderly and disabled, they are also used in the health sector for applications such as heart rate, respiratory rate, and body movement monitoring. Micro-Doppler radar provides more sensitive and customized information than other radar systems, such as conventional Doppler or imaging radar, enabling the development of new applications in security, health, and other industries [1].

Micro-Doppler radars are used in these areas because of their advantages over cameras and other types of sensors, such as their penetration capabilities, which make them advantageous for use in obscured environments. This allows them to be used through walls, smoke, fog, and so on. Radar can used in all weather conditions. This gives them advantages over cameras in foggy environments and in the rain for their use in surveillance applications and automobile industry applications. Radars also allow for surveillance without the invasion of the privacy of the targets. This makes them better candidates for elderly care. Also, radars are much more robust in their use in changing light conditions, which is an advantage for elderly care and surveillance applications [2,3].

Artificial Intelligence (AI) technology provides significant advantages in areas such as radar system signal processing and data analysis, automatic target recognition, advanced adaptation and learning, cyber security, resource optimization, and integrating new technologies. AI-based radars improve target detection and classification accuracy, optimizing energy and bandwidth usage and providing better protection against cyber attacks. In addition, AI enables the continuous improvement of radar and gives us the ability to respond better to changing situations, providing more effective and flexible solutions in security, health, and other areas [4].

Here, we present a brief summary of the literature in this field.

**Feasibility Phase:** Studies of radar-based human recognition techniques began after 2005. Lai was one of the first studies on human recognition using radar [5]. Subsequently, several studies were conducted on the feasibility of this task. These studies discuss the feasibility of human–animal separation [6], human gait analysis, and behavior analysis.

**Classical AI Applications:** Once feasibility discussions were completed, experimental applications emerged in the literature. This type of research becomes more common after 2012. In the earlier stage of this research, more classical Artificial Intelligence (AI) techniques were used, such as Support vector machine (SVM), Naive Bayes (NB), Artificial Neural Network (ANN), and Gaussian Mixture Models (GMMs). One of the earliest studies on classical AI applications was conducted by Damarla et al. The authors were interested in distinguishing between human and horse signals in their study [7]. In this experimental study, the authors conducted a data collection phase. To collect the data, people and horses were allowed to pass in front of a Doppler radar. The collected data were processed using two different classification algorithms. These classification algorithms were implemented for high and low SNR values.

**Deep Learning Applications:** After 2018, the number of deep learning-based applications of AI increased, and classical AI applications decreased, as deep learning-based classifiers are usually more robust and have a more comprehensive range of applications. In an earlier study in this area, Gurbuz and Amin [8] investigated deep learning techniques in data received from a Doppler radar. This study focuses on scenarios that may be experienced in nursing homes. Therefore, the selected environment was an indoor environment, and the scenarios included walking in a wheelchair, limping, walking with a cane, walking with a walker, falling, using crutches, and crawling.

**Security of the Applications:** In radar systems, there are two primary types of cyber attacks: Denial of Service (DoS) and Spoofing. DoS attacks are performed by transmitting noise or adversarial signals to prevent the victim radar from detecting the targets. Spoofing attacks, on the other hand, are more complex. They aim to make the victim radar detect ghost objects, prevent the detection of specific targets, or change the information about the targets. In this respect, detecting spoofing attacks is considerably more complicated than detecting DoS attacks.

A study on this was performed by Liu et al. [9], who developed an anti-velocity jamming strategy for Pulse Doppler (PD) radars to detect moving targets when they are under a digital radio frequency memory (DRFM) jammer attack.

**Figure 1:**
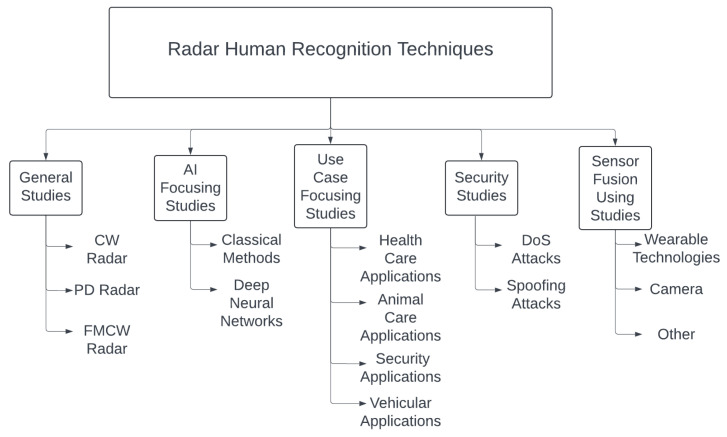
Taxonomy of Research on Radar Human Recognition Techniques.

This study evaluated the general state of radar-based human detection technology, related literature, and state-of-the-art methods. This study aimed to provide guidelines for new research in this area. Therefore, this study provides a general overview of the relevant literature, provides the reader with a general taxonomy of the literature, as shown in Figure 1, and highlights the historical development of the literature. This paper is structured as follows: First is a General Studies Section 2, which consists mainly of earlier articles in the field, followed by a Radar Types Section 3, which gives a general overview of the research on the feasibility of different radar types. The AI-Focused Studies Section 4 relates to AI techniques. The fourth section is about Use Case-Focused Studies, Section 5, which is grouped into physical security applications, the healthcare industry, nature, animal care applications, and automotive industry applications. The sixth section discusses Security Studies, Section 6, including DoS and spoofing attacks. The subsequent section is concerned with sensor fusion techniques, Section 7. In this section, we discuss the details of the sensor fusion techniques applied to micro-Doppler radars. The final section is reserved for Suggestions and redirects the reader to Section 8, discussing our suggestions for extending the literature on this subject.

## 2. General Studies

The studies listed in this section were typically conducted earlier in the field. These studies are generally interested in the feasibility of using micro-doppler radars to recognize human activities. Lai [5] conducted one of the earlier studies in this area. Lai investigates the feasibility of using random noise micro doppler radar to monitor people inside buildings. This study can also be classified as a security use of micro-doppler radar because it concerns the surveillance application of the tool. In his doctoral thesis, Lai investigated the possibility of observing the inside of a [5] wall using a random noise radar. In the experimental study, this process was performed with a high performance.

Tahmoush and Silvous conducted an early study of this area. In 2009, the authors focused on human and animal discrimination using doppler radar signals [6]. In this study, simulated signal outputs were used instead of the experimental setup. The results of this study demonstrate that this process can be performed feasibly, and features such as the trunk, swinging arms, and feet can be extracted from the signals. As a continuation of their research, the authors investigated the importance of Angle, Amplitude, Pulse Repetition Frequency (PRF), and illumination in micro-doppler radars [10]. This study showed that the angle of motion from the radar point of view is one of the most critical factors.

Bryan et al. discussed defining human behavior using a doppler radar trace [11]. Using the SVM developed in this study, walking, running, turning, punching, jumping, sitting, standing, crawling, and standing movements were defined. The necessity for further development of this pioneering work in the field is emphasized in the Results section. In particular, the shortcomings the authors emphasize the most are the necessity of making movements opposite to the radar and the decrease in performance in the horizontal area.

Gürbüz et al. investigated simulated radar signals [12]. This study evaluated the movements of 16 people running and walking against radar at an angle of 90 degrees. Owing to the small dataset size, conducting this study with larger datasets would provide a more precise evaluation of the results.

Narayanan et al. [13] studied a 6.5 Ghz micro-doppler radar trace of 18 human activities. They learned several features and inference methods to classify these movements correctly.

Chenye Li’s master’s thesis includes a gait analysis study using ultrasonic sensors [14]. Li used sensors based on the Doppler effect of ultrasonic gait on sound signals. Although the environment used in this study consisted of sound signals instead of radar signals, the main active factors were considered owing to the Doppler effect. This is the reason this study is discussed in this paper.

### Traditional Techniques

In the early stages of this research field, statistical methods were used to identify and classify human activity. Although studies using statistical methods are limited in the literature, we address the most important ones in this subsection. Using only statistical methods was insufficient for the real-life application of these technologies, but they demonstrated great potential in micro-doppler signature analysis.

Chen et al. described and demonstrated micro-doppler effects. This study investigates micro-doppler effects on mathematical foundations, creates simulated effects, and validates simulations through experiments [15]. As a continuation of that study, Chen used Independent Component Analysis (ICA) for gait analysis of a simulated data set [16]. This study gives all the basic components of human gait activity. Classifier details are considered beyond the scope of this study.

Zhang et al. proposed Hough Transform for a moving target classification preprocessing method [17]. The Hough Transform is a robust method in a low SNR environment. This ability to reduce noise makes the Hough Transform a promising preprocess method for noise reduction.

Tahmoush and Silvious [18] examined gait radar images captured from the front. The spectrographic feature extraction images were first applied to the fourier transform. Foot, arm waving, and body line extractions were performed. This study is important because it is one of the first to show that gait analysis can be performed on radar images.

## 3. Radar Types

Different types of radar can be used in human recognition systems. Therefore, articles based on various radar types were reviewed. Different types of radar have unique abilities and disadvantages. In addition, depending on the radar type, data-type generation patterns vary. Therefore, data processing and the nature of spoofing and jamming-like attacks on radars are changing.

### 3.1. Non-Modular Continuous Wave Radar

Unmodulated Continuous Wave (CW) radars are continuously broadcast at a specific frequency to detect targets [1]. The basic working principle of CW radar is based on the continuous transmission of radio waves at a specific frequency. These waves were directed towards the desired area to be observed. The part that hits the target from the waves entering the relevant area is then reflected. The receiver antenna detects returning waves and obtains information regarding the target in this manner.

**Figure 2:**
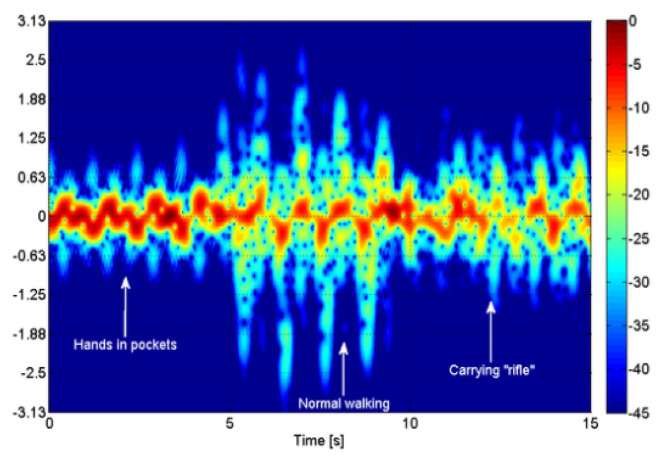
Spectogram analysis of persons walking hands in pocket, normal walking and carrying rifle [19].

Fioranelli et al. conducted several studies using a MISO-CW radar. Five of these studies determined whether a walking person was carrying a rifle [19,20,21,22,23]. These studies distinguish a person walking with a rifle depending on hand shaking while walking. These properties are demonstrated in Figure 2. Depending on this property, the real-life applications of algorithms may be hindered. Because metal objects have a superior reflective power of the RF signal when compared with the human body, this could be another property that can be exploited when classifying armed targets.

Studies have also been conducted by similar teams to identify limping animals [24,25,26]. These studies depend on similar hardware such as CW radar. The studies mentioned above concern animal care applications of CW radar, as they detect horse, cow, and sheep lameness to diagnose animals.

### 3.2. Pulse Doppler Radar

The PD radar operates by transmitting radio waves intermittently. Each of these broadcast groups is called a beat. PD radar can measure the speed and distance of moving and stationary targets in a controlled area. The return times of the pulses were measured to determine the distance between targets. The speed of the target is determined from the frequency shift of the transmitted wave. One of the most important advantages of the pulsed doppler radar is its ability to detect stationary targets. Another essential feature of these methods is their high resolutions. Consequently, PD radars can measure the speed and distance of targets with high precision.

Studies on the use of PD radars for human activity recognition are limited. Gurbuz et al. compared three radar types and sonar for indoor activity recognition. This study considered the SNR ratios, pricing, signal quality, and classification efficiency. Their study demonstrates that better alternatives exist in several respects. However, there may be limited studies on these factors in this area. Two studies have been conducted on jamming effects and strategies to limit PD radar’s effectiveness [9,27]. These studies are important for demonstrating successful spoofing attacks on PD radars.

Severino et al. studied pedestrian detection algorithms using Doppler radars in automobiles. As a result of training using PD, the desired success rates were achieved [28]. The range and quality of work performed using Multi-Objective Optimization techniques have increased. However, although the obtained results reached the desired results, they were too slow for real-time application. In this respect, this study is open to future research.

Shituru et al. [29] investigated the use of a PD radar to measure the heart rate. In an experimental study using UWB pulse-doppler radars, it was observed that measuring human breathing and heartbeat using related radars is possible.

### 3.3. Frequency Modulated Continuous Wave Radars

Frequency Modulated Continuous Wave (FMCW) are a continuously broadcasting radar. The basic working principle of FMCW radars is to broadcast them by changing their frequencies in a regular pattern. Listening to feedback from this broadcast carries information about the distance and speed of the target.

The literature on the use of FMCW radars in the healthcare industry and elderly care is limited compared to that on the use of CW radars. This could be due to the sufficient ability of CW radars or the ability of FMCW radars to detect stationary objects. This may result in an unnecessary information flow when the main objective is to detect a fall. Several studies have been conducted on this topic. Bhattacharya et al. [30] developed a FMCW radar system for fall detection. The importance of this study lies in its CNN structure, that is, RadarNet. This novel radar system detects human activity using FMCW radar data. Other studies have focused on fall detection using FMCW radars [31,32], which fuse sensors with wearable sensors to detect falls more accurately.

Vandermissen et al. made an original contribution to this area. These studies were conducted to identify specific humans in indoor environments [33]. This was achieved by using a limited human database. However, even though its human database is limited, this study promises real-world applications, such as identifying people in areas with restricted access, thus limiting the options. Another study by Vandermissen is to detect hand gestures of people such as shaking, drumming, swiping, etc. [34]. The use of vehicular FMCW radars is increasing daily. Parallel to this phenomenon, studies researching the use of these radars are growing, as are the attack types and counteracting methods regarding the limited capabilities of these radars. Section 5.4 includes more details about FMCW radars.

## 4. Artificial Intelligence Focused Studies

This section is a general abbreviation for the AI techniques used in radar human recognition. The studies listed in this section generally focused on AI techniques or have compared several techniques to demonstrate their strengths and weaknesses. There is also a general description of these techniques that can help researchers to understand them, even without prior knowledge. Arık et al. [35] compared various classification algorithms by using doppler radar signals. The algorithms used in this study were Scaled Conjugate Gradient (SCG), Levenberg-Marquardt (LM), and Bayesian Regularization Backpropagation (BRB). In conclusion, the authors emphasized that these algorithms have high processing times and need to be reconsidered for use in real-time applications.

Li et al. [36] proposed a human-activity recognition method. This method is based on Unsupervised Adversarial Domain Adaptation (ADA). The proposed method was tested using a simulated dataset. The authors compared their results with those of Gradient Reversal [37], Domain Confusion [38] and ADA [39,40]. The experiments demonstrated that the new methods are superior to previous methods.

All publications focusing on AI techniques are listed and grouped in Table 1.

Arık et al. [35] compared the performances of NB and Artificial Neural network-based algorithms for the classification of circular, square, and truncated cone targets on radar. In the comparison, Gaussian, Triangular, and Epanechnikov kernels were used for NB-based classification, whereas SCG, LM, and Bayesian regulation backpropagation methods were used for YS-based classification. They concluded that the best result was obtained using a YS-based BRB algorithm.

Wang et al. developed a random-forest-based vehicle width measurement method using FMCW radars [56]. They compared this new method with Least Absolute Shrinkage And Selection Operator (LASSO), Support Vector Regression (SVR), Linear Regression (LR), Polynomial Regression (PR) based methods and demonstrated random forest-based methods superior ability.

Cao et al. used a Deep Neural Network (DNN) to classify bicycles, cars, humans, trees, and dogs using CW radar data. The radar used in this study was an FMCWradar in CW mode. Four classification algorithms were compared in this study. These are the SVM Bayes, NB, SVM, and Deep Convolutional Neural Networks (DCNN) algorithms. The selected targets for classification were distinct targets to classify. Thus, all classification algorithms were performed with 100% accuracy, except for humans and dogs [43].

Hernangomez et al. used a Convolution Neural Network (CNN) [58]. The authors of this study improved the accuracy of this method by introducing a preprocessing system before CNN. This new method is called radar-activity classification with perceptual image transformation (RACPIT).

Xu et al. [49] studied object identification using millimeter-wave radar. A 2D image was created using a mathematical model from radar data. The classification was performed using a CNN on this image. It achieved acceptable success rates for classification.

Ningbo et al. [59] created a background classification method using CNN-LeNet background classification, which is important for reducing noise and improving classification accuracy.

Dadon et al. [60] used Short-Time Fourier Transform (STFT) and Fast Fourier Transform (FFT) as preprocessing procedures to classify human presence in radar using CNN’s. The study demonstrates the rather standard use of CNN; however, the detailed data augmentation process is worth mentioning. The authors augmented the data after preprocessing the spectrogram images using six different methods. Kim et al. [55] developed a method based on the fusion of You Only Look Once (YOLO) and SVM classification algorithms for data from FMCW radars. SVM perceives people’s Boundary Box, whereas YOLO suffers. The proposed method ensured better performance results for both algorithms. Cao et al. [43] made a comparison of AI methods to be used in the classification of doppler radar traces of ground targets. In the study where the new dataset was collected, bicycle, human, car, tree, and dog tracks were classified using SVM, DNN, NB, and Support vector machine-Naive Bayes (SVM-NB) fusion methods.

Li et al. use Bi-LSTM networks to detect people falling in healthcare facilities. Thus, this study is very valuable [31]. The use of Bi-LSTM networks strengthens their capabilities in a continuous-detection environment. This is important because it eliminates the need to use sliding windows for the detection. Another significant feature of this study is the sensor fusion with IMU devices. This study proposes two sensor-fusion methods to fuse these data to increase accuracy significantly.

## 5. Use Case Focused Studies

The micro-doppler radar technology has a wide range of applications in various sectors. This section focuses on the practical, real-world uses of this cutting-edge technology in different industries, from bolstering physical security measures to enhancing healthcare provision and from improving nature and animal care to increasing safety and efficiency in-vehicle use.

Each subsection delves into a different application of micro-doppler radars; in the physical security subsection, we will explore micro-doppler radars use in the physical security sector, and the healthcare industry subsection will focus on elderly and patient care applications, the nature/animal care industry subsection will look into on details of micro doppler radars use in the presence of different animals even in the open wild areas, the vehicular use subsection will be mentioning details of use these systems on vehicles, this section will be mainly focusing on FMCW type radars.

### 5.1. Physical Security

Lai [5] investigate the feasibility of using radars in surveillance applications; thus, it may be said that one of the first research in this area concerns the security use of radar. The use of micro doppler radars for security applications has several advantages. It may be said that their energy consumption, privacy concerns, ability to do surveillance behind walls and concealed items, and ability to be used in disadvantageous weather such as fog, rain, and snowy environments demonstrate a promising potential in the field.

Studies have been conducted using micro-doppler radars in the field of security. These studies have focused on human recognition [61], identification from behind the wall [5], and understanding the state of humans carrying a weapon [19]. In addition, human-animal discrimination and human-vehicle-cycling separation studies have also been conducted. Studies have shown that it is possible to use micro doppler radars in this area [62].

Villeval et al. [61] worked on human detection using automotive standard doppler radar. The peculiarity of this study is that it was conducted in an environment where ambient noise is high, and human behavior is not very clear because it simulates a real city environment. Human dogs and vehicles were classified as successful in this study.

Fioranelli et al. [19,20,22] conducted a study to determine whether the target persons were armed. In the first article, the team worked on the detection of a single person walking from three different angles from 1 output and two input radars. In the second article, the passing of two and a single person in front of a Multiple-Input Single-Output (MISO) radar network at five different angles was evaluated. In this study, the number of people and classification of guns were performed with high efficiency using Singular Value Decomposition (SVD) and Native Bias-based classifiers. This study was extended by Patel [23] to the same context.

The research conducted in the study of Narayanan and Zenaldin [13] was on the identification and feature extraction of human movements with doppler radar. Another remarkable aspect of this study was that the effects of imaging behind the wall were investigated and included in the analyses. The study showed that, although the wall damped the signal strength, there was no significant structural change.

Li et al. [63] investigated the feasibility of detecting concealed weapon-carrying situations with FMCW radar and Doppler Range radars. No classifications were made for this study. Only walking, walking with a concealed weapon, walking with a blind cane, walking with a bag, and walking with a shovel were performed, and the doppler radar traces of these movements were examined. We concluded that this classification was feasible at the end of the study period.

Vandersmissen et al. [33] performed human classification using FMCW radar images from five different people. A related study aimed to benefit from the biometrics of human gait behavior. The most basic criticism that can be made about this study is that the number of unique individuals in the collected dataset is very small. Thus, it may be said that their study can be classified as a health sector study using doppler radars rather than security use.

### 5.2. Healthcare Industry

The aging population and increased life expectancy have increased the demand for care services for the elderly. Elderly care is about providing medical assistance but also encompasses elders’ daily needs, such as cooking, bathing, dressing, etc. Given the state of elderly care systems, it is vital to track the essential signals of elders and detect important events such as falls. However, another important factor to be considered in the installation of this warning system is the privacy of private life. For this reason, the installation of camera systems in rooms is an undesirable option. Radar-based technologies have gained advantages as an alternative. In these areas, doppler radar provides less intrusive and more privacy-respecting alternatives [11,64].

Sasakawa et al. [65] investigated the use of CW microwave Multiple-Input Multiple-Output (MIMO) radars for human recognition. In this study, eight registered subjects and four unregistered subjects, around which there were many radars, breathed. This study, which shows that the performance ratio increases with the size of the radar network used, claims that an 8 × 8 matrix can provide a 100% performance.

Li et al. conducted a study on the combined analysis of doppler radar signals and wearable wristbands [31]. As a result, a double-layered Bi-Directional Long Short Term Memory (Bi-LSTM) network and a fusion technique were developed. The motion analyses performed using this fusion increased. The movements of walking, standing, standing, picking up an object, and drinking water were measured with a success rate close to 90%.

Yin et al. used [66] UWB radars to distinguish between standing humans and rabbits. In this study, it was shown that, after separating the breathing behaviors of humans and rabbits, they could be differentiated from the energy levels. Bhattacharya et al. [30] designed a radar system for detecting falls. The received data were classified using CNN.

Islam et al. [42] attempted to capture human breathing and heartbeat using 2.4 GHz Doppler radars. The classification using SVM was similar to the true value, with an accuracy of 92%.

Sang and Kang [67] used SFSK and FSA radar signals to detect standing humans. This study showed that standing individuals can be detected by evaluating their breathing movements.

An et al. [68] conducted a study on motion detection behind walls. They defined these movements as walking forward, walking backward, sitting, getting up, crawling forward, crawling back, falling forward, and falling back. Four different classification methods were used in the first study, and six different classification methods and two different radar systems were used in the second study.

Hamalainen et al. [54] developed a motion detection application using FMCW radar for elderly care homes. In this study, it was noteworthy to examine coughing movements and breathing.

Schooley and Hamza [69] experimented with a 77 Ghz CW radar. This study is mainly concerned with the effects of different approach angles. The authors propose a method to overcome the challenges associated with these effects. This research was supported by experimental results.

Li studied the definition of daily activities for nursing homes [32] in his doctoral thesis. In this study, sensors, such as wearable gyroscopes and glsFMCWradars, were used. With these sensors, classification accuracy is increased by the fusion of radar data in various layers.

Saho et al. [45] performed person identification using a 24 Ghz micro doppler radar. This study was based on sitting and standing movements. Ten people were used in the study dataset; therefore, the dataset could be considered narrow in terms of measuring the feasibility of applications in large areas. However, it should be considered sufficient to demonstrate its use in areas such as nursing homes and private facilities, where general entrance and exit are limited. The classification algorithm was used as CNN. Gürbüz et al. [46] investigated the feasibility of using 5.8 GHz PD Radar, 10 Ghz CW Radar, 24 Ghz CW Radar, 40 kHz CW Sonars in elderly care homes. For this purpose, he classified them as limping, walking with a cane, walking with a walker, or moving with a wheelchair. The NB classifier is used in this study. Consequently, because the range advantage of radar systems disappears in closed environments, it has been determined that sonars may be more suitable in such environments. In this direction, the authors considered factors such as the higher resolution of the sonar owing to the speed of the sound waves and the fact that it was not affected by the electromagnetic noise of the environment.

A distinct study in this field is done by Maclaughlin et al. [70]. This study concerns American Sign Language (ASL) recognition with radars. This study is worth mentioning because of the complexity of the target activity. The method used is to create a spectrogram image of activities and then use CNN to recognize their differences.

### 5.3. Animal Care

Micro-doppler radars can also be used for nature tracking or animal care. These radars have been used to warn drivers of deer on roads passing through forested areas in Canada. Understanding the limping movement of animals, such as cows and horses, has been suggested for animal care.

Shrestha et al designed a system that uses doppler radar to detect the limping of horses [24], and cows [25]. For classification, experimental validation was performed using SVM and K-Nearest Neighbors (KNN). As a continuation of these studies, Sharesta defined the movements of walking, sitting, standing up, picking up objects from the ground, drinking, and falling within the scope of his doctoral thesis [57]. In this study, Bi-LSTM machine learning techniques were used.

Huijser et al. [71] measured the reliability of FMCW radars developed to measure the occurrence of large animals such as deer on the roads. In this study, the sensors left in the selected area have been working for years, and their reliability has been measured.

Fioranelli et al. [26] studied the use of FMCW radar for lameness detection in cows and sheep. This article mentions that an experimental validation study of 51 cows and 75 sheep can accurately classify 80% of cows and 90% of sheep. Although KNN remained weaker than the two classification algorithms used, the results stated in the previous sentence were obtained using the NB classifier.

Dadon et al. [60] developed a new CNN method to distinguish between humans and animals. The MAFAT dataset, which contains radar images of humans and animals, was published in 2020 by the Israeli Ministry of Defense.

### 5.4. Vehicle Use

The use of FMCW radar is very common in vehicles, particularly in Advanced driver-assistance systems (ADAS) and autonomous vehicles. This is because related technologies have various advantages over alternatives such as Lidar and Camera, which can be considered competitors, or they are desired to be used in sensor fusion together with them. The related advantages are that FMCW radars provide better efficiency in target tracking, target speed detection, distance measurement, bad weather conditions, bad lighting conditions (asymmetrical illumination worse than low illumination), FMCW radars.

Vehicular use of doppler radar systems is crucial for ADAS. Typically, these systems use FMCW radars. These systems are vital components of the automated driving systems. Thus, errors in designing and securing may be life-threatening.

Articles focusing on this type of use have generally classified other vehicles on the road, such as detecting classification and understanding roadside pedestrians [72]. Nazer [73] surveyed the use of microwave radar systems in human identification and classification. Updating the survey prepared in 2017 has contributed to the literature.

Zabalsa et al. proposed a classification method for micro-doppler radars. In this method, the time-dependent average of the STFT signal is obtained, and feature extraction is performed using Principal Component Analysis (PCA). At the end of the feature extraction, classification is made with the help of SVM. The paper is important because it is experimental and works on embedded systems [74].

Tahmous and Silvious [6] investigated the feasibility of using doppler radar to differentiate between humans and animals. Although no classification was performed in this study, no classification was performed. Instead of mentioning the feature extraction methods, the authors mentioned how these features can be extracted from the spectrogram.

Lang et al. [75] prepared a large-scale survey on the use of machine learning techniques in radars. In their study, they also mentioned micro-doppler radars.

Allipi et al. [76] investigated human presence using Radio Tomographic Imaging (RTI) techniques. This study is in a special position in terms of being performed outdoors. In the literature, human presence measurement studies with RTI are generally performed using RSS measurements and indoor environments. This implies the need for related studies, such as those on rain. It is highly affected by external environmental factors. However, owing to the cumulative RSS-based technique developed by Allipi et al., this measurement can also be performed in an external environment.

Hyun and Jin [77] developed a human- and vehicle-classification algorithm for FMCW radars. In this study, a feature extraction mathematical modeling method was used, and the success rate was reported to be 90%.

Shi et al. [78] simulated the doppler radar traces of movements in the MoCap database. No classification was made as a result of the study using 19-point sensitivity in 3D modeling.

Li et al. [44] conducted a study on hand gesture classification. An SVM was used to classify type movements. Li et al. [27] developed a method based on expert opinions to evaluate the mixing techniques of pulsed doppler radars.

Fairchild and Narayanan [79] investigated the feasibility of using two different doppler radars to classify human movements. Both s-band and mmWave radars were able to classify breathing, swinging arms, picking up objects from the ground, and standing up from behind a wall with high accuracy with high accuracy. During this classification, the mmWave radar was more successful.

Vandersmissen et al. [34] used deep convolutional neural networks to classify six activities and six events. The dataset created by the study team included 3852 entries. With this feature, the overall classification performance of this study was greater than 90%. This performance is impressive because this study used one of the largest datasets in the literature.

Lee et al. [48] developed a new feature for human vehicle separation. SVM training was conducted using the Root Radar Cross Section (RRCS). Classification using the FMCW radar has been shown to work with a 90% success rate.

Gao et al. [47] conducted a series of experiments by placing an FMCW radar system on a vehicle. In these experiments, fixed objects and passengers were recorded in a parking area on the road. These imported records were classified using the DT Baseline and CDMC algorithms.

Bilik et al. [80] prepared a general review article on the use of radar in vehicles. In this study, factors such as multipath, clutter, and scene variety, which are specific problems in automotive use, were examined.

Rizik et al. [51] worked on CNN training so that 24 Ghz FMCW radars could distinguish between people and vehicles. Transfer learning was performed to overcome the inadequacies of the dataset, which used images captured with a camera. For this purpose, a radar spectrogram was transformed into a picture and processed. Yang calculated the target incidence angle for CW doppler radars in his work. He also measured the accuracy of the mathematical model he presented with the study he supported experimentally [81]. In the next phase of the study, he included the range and angle of incidence in his calculations [82].

Vu et al. [83] prepared a comparison article on attacks on the FMCW radars used in vehicles. In this study, eight attack types were compared.

Wang and Hu [56] developed a system for measuring the vehicle width using FMCW radar. Reliable results were obtained in the system based on feature extraction using a random forest from the data passed through the 3D FFT.

Shen et al. [84] approached the issue of autonomous vehicle safety from a semantic perspective and systematically evaluated it. In a related study, six open points were determined and evaluated in the relevant area, as well as the attacks and countermeasures to which the radars used in the vehicles were exposed.

Ulrich et al. [50] designed a new DNN for object identification using an FMCW radar for use in vehicles. This DNN, called DeepReflect, is more successful than CraftedForest and GrindCNN.

## 6. Security Studies

Jamming and spoofing attacks on radar systems can significantly affect the reliability and accuracy of the radar. Jamming attacks are carried out by emitting strong radio waves in the frequency range used by radars to disrupt and block radar signals. Such attacks make it difficult for radar to detect and track targets, thereby reducing their effectiveness and reliability. Jamming attacks pose significant security risks to the military and civilian radar systems. In military applications, jamming attacks can prevent intruder detection. In the healthcare industry, although these attacks do not intentionally target radar, some sources may create unintentional jamming effects.

However, spoofing attacks aim to hide the location and characteristics of real targets by sending false signals to deceive the radar systems. Such attacks are carried out by spoofing radar signals or sending misleading information to the radar systems. Spoofing attacks can cause radar to make inaccurate decisions and fail to detect targets. In military applications, spoofing attacks can obscure the true location of enemy targets and prevent friendly forces from taking effective actions against enemy targets. In civilian applications, spoofing attacks target automobile FMCW radars to misjudge the true position and speed of other vehicles and force them to crash. Such crashes can be fatal.

The following measures can be taken to combat jamming and spoofing attacks and make radar systems more resistant to such attacks:

**Frequency hopping radar technology:** By using the frequency hopping features of radar systems, you can reduce the impact of jamming and spoofing attacks. Frequency-hopping radars switch between different frequencies quickly, making it difficult for attackers to block radar signals or effectively send spoofed signals.

**AI and machine learning:** AI and machine learning algorithms can be used to analyze radar signals and in target detection and recognition processes. These technologies can be used to isolate real targets from bogus signals and to reduce the impact of jamming attacks.

**Signal processing and data analysis:** Advanced signal processing and data analysis methods can help radar systems become more resistant to jamming and spoofing attacks. These methods increase the accuracy and reliability of radar systems by enabling more accurate and faster analysis of actual targets and false signals.

Improvements in the signal-processing and data-analysis capabilities of radar systems, frequency-hopping radar technologies, and AI-assisted detection and recognition algorithms can be used to counter jamming and spoofing attacks. These approaches can help radar systems become more resilient to attacks and provide more reliable and accurate results. Effective measures against jamming and spoofing attacks and making radar systems more resistant to such attacks will help reduce security risks and prevent potential damage.

**Multi-sensor integration:** Integrating different types of sensors (e.g., optical, infrared, acoustic) with radar systems can provide an additional layer of protection against jamming and spoofing attacks. This ensures that radar systems can perform effective target detection and tracking while maintaining the accuracy and reliability of data obtained using other sensors, even if they are vulnerable to one type of attack.

**Collaborative radar systems:** Collaboration and data sharing between multiple radar systems provide a better defense against jamming and spoofing attacks. Collaborative radars can work together to detect and track targets, achieving effective target acquisition and tracking, even in the event of a single radar system failure. Consequently, effective measures against jamming and spoofing attacks are essential to maintaining radar systems’ security, reliability, and effectiveness. Furthermore, these measures and strategies will increase radar systems’ resilience against such attacks, helping minimize potential damage.

All publishings focusing on Spoofing techniques are listed and grouped in Table 2.

### 6.1. DoS Attacks

One type of attack is jamming. Jamming attacks are generally based on broadcasting noise from the same channel to prevent the radar system from operating [98]. Attacks made in this way can cause vehicles to blind FMCW radars, see fake objects, or seriously reduce the detection range and reliability of existing targets. This can ensure that autonomous vehicles are not forced into an accident [72].

Zao et al. [99] developed a jammer against CW land observation radar for tracked vehicles. Using this method, which is based on the physical geometry of the pallets, a high-resolution aggressive signal can be produced based on the results obtained from the simulations.

Lazaro et al. [95] designed a backscatter tag to attack an FMCW radar with spoofing. Like passive RFIDs, the device is designed to respond to a spoofing signal when it detects a 24 GHz signal.

Fioranelli [87] designed a jamming attack on CW radar. This study aimed to create a classification system that works with corrupted data. To achieve this, they used NB, KNN SVM-Multi, and SVM-Mono classifiers. The best results were achieved using SVM multiclassifiers.

Patel [88], who worked with Fioranelli et al., published a fusion sensor fusion technique for counteracting the effects of jamming. Their architecture is based on ALEXNet (ALEXNet) [100]. The nodes have 14 classes, each sharing their results with confidence metrics. The final result is selected based on the most confident prediction of this notion.

### 6.2. Spoofing

Spoofing attacks send countersignals to deceive the radar systems. Radar-spoofing attacks can send back radar signals in different forms, causing false targets to appear or real objects to disappear. Spoofing attacks are more sophisticated than jamming attacks and are more difficult to detect and countermeasure.

For radar, spoofing attacks are performed in the physical layer by sending a signal that is similar to the one that must be returned in response to the radar signal. The purpose of these attacks may be to add an object that does not exist, hide the existing object, or change the quality (speed and shape) of the existing object.

Rastogi et al. [101] developed a Dempster-Shafer Theory (DST) belief intrusion detection system for autonomous vehicles against spoofing attacks that may occur on radar. Guendel [102] did a study concerning the classification of daily activities with FMCW and CW radars within the scope of his master’s thesis. In this study, in which many radars placed in a closed environment could be used simultaneously, a state machine was used to make sense of motion transitions.

Xu et al. [97] investigated spoofing attacks in autonomous vehicles and developed countermeasures against them. In this context, radar types used in vehicles were discussed and examined in this study.

Rodriguez et al. [86] performed a deception attack on a micro-doppler radar. This attack is the only example found in the literature review of this area. In a related attack, an attack was carried out by imitating this feature of the human against systems that defined humans based on the characteristics of the cardiovascular system.

Ilioudis et al. [103] developed a jammer Electronic countermeasure (ECM) for the self-defense of microdrones. The feasibility of this ECM was demonstrated through simulations based on the POFACETS and mathematical models.

Shenoy et al. [52] developed a system to prevent people inside a house from being tracked through a wall using FMCW radar. The system performs spoofing attacks on the tracking radar by reflecting the signals of the FMCW radar based on certain parameters. Thus, it acts as a means of protecting people. The system was experimentally validated, and the results were confirmed.

Chipengo et al. [53] simulated FMCW radar images of the movements of vehicles, dogs, pedestrians, and cyclists. This study obtained a high-performance network by training the simulation results using CNN. The results section of the study emphasizes that more reliable training can be performed owing to the ease of generating data with the help of simulations.

Ordean and Garcia [90] investigated spoofing attacks on FMCW radar. At the end of the experiment, which was also tested experimentally, attacks, including ghost object creation, were successful.

Nallabolu et al. [93] studied spoofing attacks on FMCW radars. This study ensured that the human target, which was stopped by experimental studies, was seen as if the victim was on the radar. In the next phase of the study, ref. [94] attempted attacks that could be performed in human detection applications using FMCW radars. The attacks were as follows: imitation of human walking movement, imitation of a standing person, imitation of a human standing behind a wall, and imitation of vital features.

In Komissarov and Wool’s study, spoofing attacks were conducted on vehicle FMCW radar, and security measures were recommended against these attacks [91]. For this attack, the victim vehicle’s FMCW radar manipulates the signals through the SDR hardware located behind the attacker vehicle that passes in front of the victim vehicle. Through the signals sent in this manner, critical data such as the speed of the attacking vehicle and the distance between the two vehicles are falsely detected in the victim vehicle. In the experimental study, a “bladeRF xA4” device was used in the system where the POC study was performed. We believe the attack is significant in targeting the FMCW system in many current high-end vehicles and, therefore, ADAS systems. The last part of this article discusses the mechanisms for preventing attacks. The three proposed prevention mechanisms are as follows: (1) Camera radar sensor fusion, as in [41] or the development of a sensor fusion algorithm similar to that used in [85]. (2) The attacks are based on a hardware phase difference measurement mechanism.

Sun et al. [72] 2 attack and defense methods against millimeter-wave radar (mmWave) radars. The methods proposed in related studies were implemented and tested on an autonomous vehicle. In the experiment, the decision-making mechanisms of related autonomous vehicles were deceived in this manner, and the system was verified. Five scenarios were tested in the experiment. In the first scenario, a ghost object was created before a stationary vehicle, and the vehicle in the red light moved later. In the second scenario, hard braking and threatening passengers were attempted by creating a ghost object in front of the moving vehicle. In the third scenario, an obstacle was created in front of a moving vehicle, and the victim’s vehicle changed lanes accordingly. In the 4th scenario, the victim vehicle is first forced to change lanes by creating a ghost object in the front. In the 5th scenario, the curve control system of the non-autonomous victim vehicle was exposed to the attack, and sudden braking of the vehicle was provided with the help of the ghost object created.

## 7. Sensor Fusion

Sensor fusion involves the combination of data from multiple sensors to provide information that is more accurate, reliable, and comprehensive than that provided by individual sensors [104]. Sensor fusion on micro-doppler radar systems generally increases accuracy and prevents cyber-attacks. Kommisarov [91] proposed a camera and FMCWradar to detect spoofing attacks. Another study was conducted by Wang et al. [105] on the sensor fusion of signals received using millimeter-wave radar with camera images. It aims to increase the accuracy of the analyses by addressing the radar signals received in the region of interest.

Li et al. [31] have developed an algorithm that recognizes the events of walking, sitting, standing, taking objects, drinking water, and falling with the data obtained from doppler radar, accelerometer, gyroscope, and magnetometer. For classification, data from the accelerometer, gyroscope, and magnetometer were combined using a single internal measurement unit (IMU), whereas data from the radar were evaluated separately. The main subject of this study was sensor fusion with deep learning using the two main data sources.

In a study by Jong et al. [41], sensor fusion algorithms were developed for doppler radar traces and camera images. Fusion techniques, particularly those designed for human identification algorithms, can be divided into three levels: data, attributes, and features. Ideal light and weather conditions were used during the tests. Expanding this study to include more challenging environmental conditions in future studies will be possible.

Another method to gain resilience against spoofing attacks is vehicle-to-vehicle communication and sensor fusion of the radars of different vehicles. Yang, etc. [85] proposes a framework for vehicle sensor fusion. The sensor fusion algorithm is a sensor-based measurement-level fusion algorithm. The algorithm depends on over half of the vehicles the attackers do not compromise. In this scenario, the system can detect and correct compromised vehicles. Further studies should be conducted to perform better in scenarios where more than half of the vehicles are compromised.

All publishing focusing on Sensor Fusion techniques are listed and grouped in Table 3.

## 8. Suggestions & Redirects

Boche et al. [106] showed that, in the context of communication systems, it is not possible to understand algorithmically whether a physical channel is jammed. This is because the related problem is the same as the halting problem. Regarding the working principle of radars, they broadcast radio frequency on a particular channel and generate data upon returning from the target. In this respect, it is natural to consider radar sensor channels to be analog communication channels. As can be understood from the above, it is impossible to comprehend algorithmically whether a jamming attack occurs on a channel in radar systems.

In the field of cyber security, it can be observed that problems that have been proven to be equivalent to the halting problem are solved with the help of CNNs [107,108]. It should be seen as a problem worth working on to understand whether radar jamming is performed in a channel in similar studies.

Generating radar spectrograms and applying CNN-based classification methods were established. The application of more regular image processing methods can be studied. Sengel et al. [109] proposed a new method to recognize fingerprints. Therefore, the application of these algorithms to spectrogram images is a promising approach.

Studies have focused on extracting features such as leg and arm movements for gait analysis. These features play a crucial role in the understanding of human motion and have numerous applications. Gait analysis capabilities enable the distinction of various human characteristics, such as sex, height, and even personal identification, because gait is considered a unique biometric feature.

In security and surveillance applications, gait analysis can aid in identifying suspects or individuals of interest without the need for facial recognition, thereby ensuring non-intrusive monitoring. In healthcare, gait analysis can be employed to detect and monitor mobility disorders, evaluate rehabilitation progress, and assess treatment effectiveness. Furthermore, in sports science, gait analysis can help athletes and coaches understand an individual’s performance, identify areas for improvement, and prevent injuries by highlighting improper movement patterns. Overall, the ability to analyze gait patterns and extract meaningful features has significant implications in various fields, enhancing our understanding of human motion and providing valuable insights for different applications.

With the widespread use of such sensors, especially in the security field, cyberattacks on such sensors are predicted to increase. It has been observed in the literature that publications in this field are limited and one-dimensional [86]. There are no studies in the literature on the introduction of humans as vehicles or animals into systems that detect humans. Therefore, it was observed that a new type of deception attack should be developed for systems with human identification.

While previous studies have focused on detecting limping in animals, such as cows and horses, classifying animal species using radar technology remains relatively unexplored. Such research could greatly benefit wildlife preservation workers and conservation efforts by providing efficient and nonintrusive methods for monitoring and managing various species in their natural habitats.

By distinguishing between animal species, conservationists can gain valuable insights into the distribution, population dynamics, and behavior of different animals in a given area. This information could inform the development of targeted strategies for preserving and protecting endangered species or for controlling the spread of invasive species.

Additionally, understanding animal movement patterns and behaviors can help scientists study the impacts of climate change and human activities on ecosystems, enabling the design of better conservation policies and sustainable land management practices.

Counting people using doppler radar is a valuable feature because it can provide significant benefits in various settings, such as supermarkets, street density monitoring, and other smart city applications. People counting can contribute to the efficient allocation of resources, enhance public safety by monitoring crowd levels during events or emergencies, and support data-driven decision-making processes to create sustainable and livable urban environments.

Identifying vehicle types, such as cars, jeeps, minibusses, buses, and trucks, is a highly desirable feature of FMCW radars, particularly in the automotive industry. This capability allows for enhanced traffic management, better safety measures, and improved collision avoidance systems by providing precise information about the surrounding vehicles. In addition, it facilitates ADAS and paves the way for the development and deployment of autonomous vehicles, which rely heavily on accurate vehicle-type identification to make informed decisions and ensure safe navigation in complex traffic environments. Furthermore, this feature can aid transportation infrastructure planning, traffic monitoring, and congestion management by offering valuable insights into vehicle-type distributions and their specific behaviors on the road.

## 9. Results

This comprehensive study aims to provide researchers with a thorough review of the existing literature. This study illuminates the details of micro-doppler radar human recognition techniques and provides an exhaustive review of the subject. This study is a repository for numerous studies that have been listed, critically evaluated, and systematically classified.

The current literature is illuminated, key findings highlighted, and gaps that future studies may aim to fill are identified. It aims to present an unbiased and balanced view of the literature, considering the wide range of methodologies, theories, and perspectives employed in studies. Furthermore, we critically evaluated each study, assessed its strengths and weaknesses, and discussed.

In conclusion, this paper is a review and extensive guide to the literature on micro-doppler imaging. This is a valuable resource for researchers to assist them in comprehending the current state of the field, identifying gaps in the literature, and guiding future research in this area.

## Figures and Tables

**Table 1:** Publishings focuses micro doppler radar AI techniques.

Publishing	Radar Type	Classification	AI Technique
[41]	CW	Human Walking	CNN, LSTM
[8]	CW	Human Activities	DNN
[24,25,26]	CW	Animal Lameness	KNN, NB
[42]	CW	Human Respitorary system	SVM
[43]	CW	Bicycle, Car, Human, Tree, Dog	DNN, SVM, NB
[30]	CW	Human Fall Detection	CNN
[44]	CW	Hand gestures	SVM
[45]	CW	Human Activities, Identification	CNN
[46]	CW, PD	Limping, cane, walking, wheelchair	SWM, NB
[20]	CW, MISO	Human With And Without Rifle	PCA, SVD
[19,22,23]	CW, MISO	Human With And Without Rifle	NB
[47]	FMCW	Multiple Pedestrian and Object	DT, CDMC
[31]	FMCW	Different Human Activities	Bi-LSTM DNN
[28]	FMCW	Human Detection	GA, RS, SVM
[48]	FMCW	Human Vehicle Cyclist	SVM
[49,50,51]	FMCW	Human Vehicle Cyclist	CNN
[52]	FMCW	Human Trajectories, breathing	Bi-LSTM
[53]	FMCW	Car Pedestrian Cyclist Dog	CNN
[54]	FMCW	Falling, Sitting, Standing	KNN
[55]	FMCW	Vehicle, Pedestrian	SWVM-YOLO
[56]	FMCW	Vehicle Width	RF
[34]	FMCW	Hand and Gait Activities	LSTM, CNN
[32,57]	FMCW	Human Activities Indoors	Bi-LSTM

**Table 2:** Publishings about spoofing techniques.

Publishing	Radar Type	Classification	Spoofing Technique
[85]	NA	Vehicle, Speed	Sensor Level Attack
[9]	PD	Target	Jamming Signal
[86]	CW	NA	Backscatter Attack
[87,88]	CW	Human walking with Rifle	Jamming Attacks
[89]	FMCW	NA	Replay attack
[90]	FMCW	NA	Adversary Signal
[91]	FMCW	Vehicle, Speed	Backscatter Attack
[92]	FMCW	Vehicle, Speed	Adversary Signal
[72]	FMCW	Vehicle, Speed	System Level Attack
[93,94]	FMCW	Walking, Stationary Human	Adversary Signal
[95]	FMCW	Generic Object	Backscatter Attack
[96]	FMCW	Generic Object, Distance	Replay attack
[52]	FMCW	Human Trajectories, breathing	Eavesdropping
[84,97]	FMCW	Object	System Level Attack
[98]	FMCW	Object/Vehicle	Sensor Attacks

**Table 3:** Publishings about micro doppler radar sensor fusion techniques.

Publishing	Radar Type	Classification	Fusion Technique
[85]	NA	H∞	Radar-Radar
[41,105]	CW	CNN, RNN, LSTM	Camera
[46]	CW, PD	SWM, NB	CW Sonar
[91]	FMCW	NA	LiDar/Camera
[31]	FMCW	Bi-LSTM	Wrist IMU, Ankle IMU
[55]	FMCW	SWVM-YOLO	YOLO-SVM
[34]	FMCW	DNN, LSTM, CNN	Camera
